# Amplicon –Based Metagenomic Analysis of Mixed Fungal Samples Using Proton Release Amplicon Sequencing

**DOI:** 10.1371/journal.pone.0093849

**Published:** 2014-04-11

**Authors:** Daniel P. Tonge, Catherine H. Pashley, Timothy W. Gant

**Affiliations:** 1 Centre for Radiation, Chemical and Environmental Hazards, Public Health England, Harwell Campus, Didcot, Oxfordshire, United Kingdom; 2 Department of Infection, Immunity and Inflammation, Institute for Lung Health, University of Leicester, Leicester, United Kingdom; 3 Faculty of Computing, Engineering and Sciences, Staffordshire University, Stoke-on-Trent, United Kingdom; California Department of Public Health, United States of America

## Abstract

Next generation sequencing technology has revolutionised microbiology by allowing concurrent analysis of whole microbial communities. Here we developed and verified similar methods for the analysis of fungal communities using a proton release sequencing platform with the ability to sequence reads of up to 400 bp in length at significant depth. This read length permits the sequencing of amplicons from commonly used fungal identification regions and thereby taxonomic classification. Using the 400 bp sequencing capability, we have sequenced amplicons from the ITS1, ITS2 and LSU fungal regions to a depth of approximately 700,000 raw reads per sample. Representative operational taxonomic units (OTUs) were chosen by the USEARCH algorithm, and identified taxonomically through nucleotide blast (BLASTn). Combination of this sequencing technology with the bioinformatics pipeline allowed species recognition in two controlled fungal spore populations containing members of known identity and concentration. Each species included within the two controlled populations was found to correspond to a representative OTU, and these OTUs were found to be highly accurate representations of true biological sequences. However, the absolute number of reads attributed to each OTU differed among species. The majority of species were represented by an OTU derived from all three genomic regions although in some cases, species were only represented in two of the regions due to the absence of conserved primer binding sites or due to sequence composition. It is apparent from our data that proton release sequencing technologies can deliver a qualitative assessment of the fungal members comprising a sample. The fact that some fungi cannot be amplified by specific “conserved” primer pairs confirms our recommendation that a multi-region approach be taken for other amplicon-based metagenomic studies.

## Introduction

The fungal kingdom is highly diverse and includes taxa from numerous different ecological niches with varied life history strategies and morphologies, ranging from unicellular yeasts and microscopic moulds to large mushrooms. The number of formally described fungal species is over 74,000 with greater than 12,000 named species in the British Isles alone, however, estimates of the number of species globally is believed to be at least 1.5 million, but probably as many as 3 million [1], [2]. Spores are one of the mechanisms used by fungi for dispersal, most of which are wind-borne and vary greatly in size, morphology and method of release; with both passive and active spore liberation methods. Fungi are an essential component of the ecosystem and have major economic value, both positively (commercial exploitation for drugs, food-stuff, fuel, pesticides etc. [3]) and negatively as plant and animal pathogens, and play an important role in human health as allergens, pathogens and commensals.

Traditional methods for identifying microscopic fungi and airborne fungal spores rely on culture or morphological identification by microscopy. Culture-dependant methods inevitably underestimate diversity, and grossly bias studies towards fungi that can be cultured on generic fungal growth media. Estimates suggest that of the known fungi, only 17% can be readily grown in culture [3] and of those many will produce only sterile mycelium (Bridge and Spooner, 2001). In clinical studies, reliance on culture can miss potentially important fungi [4]. Microscopy is less prone to bias; however, many spores cannot be distinguished from each other based upon their morphology alone. Of the 40 or so fungal categories that can be recognised, some can be classified to genus, a few to species, but many have to be recorded in groups with similar characteristics [5].

The development of molecular techniques, that utilise polymerase chain reaction (PCR) to amplify regions of the fungal genome thought to be specific to a particular species, provided the first culture-independent means of detecting microorganisms. Amplicon-based fungal metagenomic studies target various regions of the nuclear ribosomal operon (rDNA); which is present in multiple copies, contains both highly conserved and variable regions, and for which numerous universal primers exist. The fungal ribosomal operon consists of the small subunit (SSU or 16S/18S), and the large subunit (LSU or 23S/25S/28S), separated by an internal transcribed spacer (ITS) region (comprising two sections, ITS1 and ITS2) that bracket the conserved 5.8S. Three prime of the LSU is the 5S ribosomal gene flanked by two intergenic spacer regions (IGS1 and 2 respectively) [6].

Early culture-free molecular studies of fungi [7]–[9] required the amplification of fungal DNA using conserved oligonucleotide primers, and utilised the sequencing method of Sanger to determine the sequence of the resulting amplicons [10]. Sanger sequencing provided the long read length (∼1000 bp) and base-calling accuracy required for such analyses, however was unsuitable for the generation of sufficient sequencing data (termed sequencing depth) to resolve complex microbial systems commonly associated with environmental sampling, in a reasonable timeframe. The introduction of next generation sequencing revolutionised microbial metagenomics by providing much greater sequencing depth. This however tended to be at the expense of read length, with some next generation platforms generating sub-100 bp reads. The early fungal amplicon-based metagenomic studies relied on expensive 454 sequencing to enable the generation of reads long enough for species discrimination [11]–[13]. However the recent availability of affordable “bench-top” machines with sufficient read length has placed the ability to generate millions of sequencing reads into the hands of small to medium sized research laboratories. Several high throughput sequencing platforms are now available, differing most significantly in the length and depth of sequence they generate, and in their “error profile”. Choice of sequencing platform therefore represents a complex decision involving read length, read depth, base calling accuracy, systematic error profile and of course economics.

During the year of 2013, a post light (proton release) sequencing platform announced the ability to sequence reads up to 400 bp in length at significant depth (several millions of reads), within a period of hours. These specifications permit sequencing of amplicons from the ITS regions 1 and 2 and the D1/D2 region of the LSU, providing the maximum sequence length for speciation, and therefore lend themselves to fungal amplicon-based metagenomic applications. In this study, we sought to validate the use of proton release sequencing data, generated on a bench-top sequencer, to analyse mixed fungal samples using two controlled populations containing members of known identity and known concentration.

## Materials and Methods

### Fungal Spore Stocks, Mock Fungal Populations and DNA Isolation

Two controlled mock fungal populations were created. The first consisted of eight species representing eight genera (abbreviated to 8GM) of common airborne fungal spores of varying size and complexity (Table 1, 8GM). The second contained twenty species (abbreviated to 20SM) comprised of closely related fungi from four fungal genera that produce similar sized single celled spores (Table 1, 20SM).

**Table 1:** Details of the two mock fungal populations.

Species (8GM)	Strain	Spore size (µm)	Spore complexity	Concentration
Alternaria alternata	EGS 35–193	25–60 ×3–3.5	Multi-cellular (up to 15 cells)	1×10^5^/ml
Aspergillus fumigatus	NRRL 163	2.5–3.5	Single celled	1×10^5^/ml
Botrytis cinerea	CABI 160282	7–14×6–9	Single celled	1×10^5^/ml
Cladosporium herbarum	NCPF 2564	5.5–23×4–6	One or two celled	1×10^5^/ml
Epicoccum nigrum	CABI 127257	15–25	Multi-cellular (up to 15 cells)	1×10^5^/ml
Fusarium moniliforme	NCPF 2865	4–7×25–50	1 to 3 cells	1×10^5^/ml
Leptosphaeria coniothyrium	CABI 52734	3–5×15–29	Multi-cellular (up to 20 cells)	1×10^5^/ml
Penicillium chrysogenum	NCPF 2715	1.8–3.5	Single celled	1×10^5^/ml

Species (20SM)	Strain	Spore size (µm)	Spore complexity	Concentration
Aspergillus flavus	ATCC 16883	3–6	Single celled	1×10^6^/ml
Aspergillus fumigatus	NRRL 163	2.5–3.5	Single celled	1×10^6^/ml
Aspergillus niger	ATCC 16888	2.5–10	Single celled	1×10^6^/ml
Aspergillus ochraceus	NRRL 398	2.5–3	Single celled	1×10^6^/ml
Aspergillus restrictus	FRR 3689	3.8–9.5×3–4.4	Single celled	1×10^6^/ml
Aspergillus sclerotiorum	ATCC 16892	2.2–3.8	Single celled	1×10^6^/ml
Aspergillus ustus	NRRL 275	3–3.5	Single celled	1×10^6^/ml
Cryptococcus laurentii	NCYC 3360	3.5–7	Single celled	1×10^6^/ml
Cryptococcus neoformans var. grubii	ATCC 208821	2.5–10	Single celled	1×10^6^/ml
Cryptococcus victoriae	NCYC 3128	3–5×2–3	Single celled	1×10^6^/ml
Cryptococcus wieringae	NCYC 3730	4–7×5–8	Single celled	1×10^6^/ml
Paecilomyces variotii	ATCC 22319	3–5×2–4	Single celled	1×10^6^/ml
Penicillium brevicompactum	FRR 862	3–4.5	Single celled	1×10^6^/ml
Penicillium chrysogenum	NCPF 2715	1.8–3.5	Single celled	1×10^6^/ml
Penicillium citreonigrum	FRR 2046	2–2.4	Single celled	2×10^6^/ml
Penicillium corylophilum	FRR 802	2.5–5	Single celled	1×10^6^/ml
Penicillium digitatum	FRR 1313	2.5–5	Single celled	1×10^6^/ml
Penicillium fellutanum	FRR 746	2.5–5	Single celled	1×10^6^/ml
Penicillium piceum	NCPF 2720	2.5–5	Single celled	1×10^6^/ml
Penicillium spinulosum	FRR 1750	3–3.5	Single celled	1×10^6^/ml

Concentration refers to the number of spores used for DNA extraction. ATCC - American Type Culture Collection, CABI - CAB International fungal culture collection, EGS - E.G. Simmons Culture Collection, FRR - Culture collection of CSIRO Sydney Australia, NCPF - National Collection of Pathogenic Fungi, NCYC - National Collection of Yeast Cultures, NRRL - Northern Regional Research Laboratory, UAMH - University of Alberta Microfungus Collection and Herbarium

Fungal cultures were grown at room temperature on potato dextrose agar (Oxoid) until confluent and sporulating. Spores were harvested by inoculating plates with 5 ml filter sterilised 0.5% Tween 80 (Sigma-Aldrich) and filtering the fungal suspension through a Whatman filter of pore size 22 µm. The concentration of suspended spore stocks was determined by counting in a haemocytometer chamber at ×400 magnification. All spore stocks were determined by microscopic analysis to be composed of >95% spores, as opposed to mycelial cells or conidiophores. Aliquots of individual spore stocks were mixed and diluted in 0.5% Tween 80 to give a final concentration of approximately 1×10^5^ and 1×10^6^ spores/ml of each strain for the 8GM and 20SM mock populations respectively (Table 1).

100 µl of the mock population spore mixtures were added to sterile 2 ml screw cap tubes containing 600 mg (±60 mg) 212 to 300 µm sterile acid-washed glass beads (Sigma-Aldrich), 400 µl of buffer AP1, and 4 µl of 100 mg/mL RNase A (DNeasy plant kit, Qiagen). These were subjected to 2 min bead-bashing (BioSpec mini bead beater-16) then incubated for 10 min at 65 °C. Total genomic DNA was extracted using the DNeasy plant mini kit (Qiagen) following the procedure reported in [14].

### Fusion PCR

Amplification of fungal ITS1, ITS2 [15] and the D1/D2 region of the LSU [16] was performed using fusion primers containing both a gene specific priming element and an oligonucleotide adapter region required for subsequent library preparation and sequencing. Forward primers were fused with sequencing adapter (A) and reverse primers with a truncated version of adapter P1 (**Table 2**). PCR reactions comprised 22.5 µl of Platinum PCR SuperMix High Fidelity (Invitrogen), 0.5 µl of 10 µM primer mix and 2 µl of fungal DNA making a total reaction volume of 25 µl. Independent PCR reactions were conducted for each of the three fungal regions using the following cycling conditions; 94°C for 3 minutes, followed by 30 cycles of 94°C for 30 seconds, 60–68°C for 30 seconds, 68°C for 60 seconds. The resulting PCR products were purified from excess oligonucleotide primers and salts using the MinElute PCR Purification system (Qiagen) and eluted in 10 µl of molecular biology grade water. Following purification, 1 µl of each resulting PCR product was analyzed by capillary electrophoresis on a DNA 1000 chip (Bioanalyzer, Agilent Technologies) to confirm the expected fragment size was obtained, and to determine the concentration. All amplicons were diluted to 26 pM in molecular biology grade water prior to library preparation.

**Table 2:** Fusion oligonucleotide primers containing target specific sequences (White, Bruns et al., 1990, Issakainen, Jalava et al., 1999; emboldened), sequencing adapters (plain text) and the Ion Torrent key sequence (underlined).

Region	Forward Primer	Reverse Primer	Tm (°C)
**ITS 1**	CCATCTCATCCCTGCGTGTCTCCGACTCAG **TCCGTAGGTGAACCTGCGG**	CCTCTCTATGGGCAGTCGGTGAT**GCTGCGTTCTTCATCGATGC**	66
**ITS 2**	CCATCTCATCCCTGCGTGTCTCCGACTCAG **GCATCGATGAAGAACGCAGC**	CCTCTCTATGGGCAGTCGGTGAT**TCCTCCGCTTATTGATATGC**	60
**LSU**	CCATCTCATCCCTGCGTGTCTCCGACTCAG **GAGTCGAGTTGTTTGGGAATGC**	CCTCTCTATGGGCAGTCGGTGAT**GGTCCGTGTTTCAAGACGG**	68

### Ion Torrent Library Preparation and Sequencing

Emulsion PCR was performed using the Ion PGM Template OT2 kit that is suitable for reads up to 400 bp in length (product code - 4479878) in accordance with the manufacturer's standard protocol (MAN0007219 Rev. 1.0) using the OneTouch 2 instrument (Life Technologies). Sequencing was performed on the Personal Genome Machine using a 400 bp sequencing kit (4482002) with a separate 314V2 chip (4482261) used for each amplicon in accordance with the manufacturer's standard protocol (MAN0007242 Rev. 2.0).

### Bioinformatic Sequence Analysis

Raw reads were first filtered to retain only those comprising complete amplicon sequence data by searching sequentially for the presence of each forward and reverse PCR primer. Forward primer sequences were trimmed using CLC Genomics Workbench version 5.0, whilst reverse primer sequences were trimmed using CutAdapt [17]. No mismatches between the expected primer sequence and the reads were permitted. Following primer removal, full length reads were processed using the USEARCH algorithm version 7.0.1001_i86linux32 of Edgar [18] as follows: (1) identical reads were dereplicated using the -derep_fulllength command, (2) reads were denoised, merging reads with up to 1% errors using the -cluster_smallmem command specifying an identity (id) of 0.99 whilst retaining cluster size information. Clusters with less than 25 identical reads were discarded, (3) chimeric reads were removed using the -uchime_denovo command, (4) reads were clustered into operational taxonomic units (OTUs) with a maximum distance of 3% using the -derep_fulllength command specifying options -id 0.97 (3%) and -centroids. Sequence data has been deposited in the NCBI sequence read archive (SRA) with the following BioProject accession number: 235189.

### Bioinformatic Mapping to NCBI Database

In order to ascertain which species each OTU represented, representative sequences were mapped to known fungal sequences deposited in the NCBI database using nucleotide blast (BLASTn) with default parameters. The following ENTREZ term was included to suppress results from uncultured/environmental samples: all [filter] NOT(environmental samples[organism] OR metagenomes[orgn] OR txid32644[orgn] OR sp.[title]). An OTU was considered to be specific at the species level (S) if only one species attained the highest bit score. The genera *Aspergillus* and *Penicillium* each contain over 150 species separated into 16 and 25 sections respectively. Species were allocated to their sections according to current classification [19]–[24] and specificity at the section level (Se) denoted when multiple species from the same section shared the highest bit score. Specificity at the genus level (G) was denoted if more than one species (of the same genus) shared the highest bit score. OTUs which spanned multiple genera were denoted as other (O).

## Results

This is the first study to validate the use of 400 bp proton release sequencing data for fungal amplicon-based metagenomic studies using two controlled populations containing 8 and 20 species respectively. The well characterised ITS1, ITS2 and D1/D2 LSU regions (**Table 2**) were targeted in both populations and a separate sequencing reaction performed for each of the 6 resulting amplicon pools (three amplicons for each of the two controlled populations).

Four-hundred base pair (400 bp) sequencing generated 747,229±61,555 (mean ± S.E.M) raw reads per sample. Following forward and reverse adapter removal, 440,552±60,168 reads representing full length amplicons (comprising the entire expected sequence) were retained for further analysis (**Table 3**). On average, approximately 60% of the raw reads obtained were found to represent full length amplicons. In general, the sequencing of shorter amplicons (for example ITS1, mean length ∼200 bp) tended to produce more full length reads than longer amplicons (for example the D1/D2 region of LSU, mean length ∼350 bp). Until very recently, proton release sequencing technology has been limited to read lengths of ≤300 bp rendering it unsuitable for comprehensive ITS2 or LSU assessment. Whilst a recent (August 2013) publication implemented 300 bp proton release sequencing for the assessment of fungal diversity in soil, this analysis was restricted to the ITS1 region due to sequence length constraints, and further considered only the first 150 bp of this region [25]. In contrast, we consider full length sequence data from three genomic regions, which provides additional genetic information for identification at the species level.

**Table 3:** Next generation sequencing read statistics.

Sample ID	Genomic Region	Raw Reads	Full Length Reads	Length (bp)	Dereplicated Unique Reads	Denoised Unique Reads	Total Reads Available
**8GM1**	ITS1	783,648	631,395	186.7	039,691	028,439	575,752
**8GM4**	ITS2	844,884	512,545	302.7	273,496	205,294	223,048
**8GM7**	LSU	600,301	284,547	345.4	147,167	099,932	138,137
**20SM2**	ITS1	870,048	525,793	207.6	071,650	035,045	192,623
**20SM5**	ITS2	865,582	436,590	316.3	246,924	161,896	454,395
**20SM8**	LSU	518,906	252,447	343.5	193,944	154,669	059,641

Raw Reads - the total number of reads generated by each sequencing run; Full Length Reads - the total number of reads representing a full length amplicon; Dereplicated Reads - the number of unique read clusters once all identical reads were collapsed; Denoised Reads - the number of unique read clusters once reads within 1% sequence identity were combined; Total Reads Available - the total number of reads (the sum of all clusters) available for further analysis.

To reduce data redundancy, all reads were dereplicated by merging identical reads whilst retaining cluster size information. In order to reduce the impact of sequencing errors on OTU formation, full length reads were denoised, merging reads with up to 1% errors whilst retaining cluster size information so that the total number of reads available for analysis remained the same. At this stage, clusters comprising less than 25 individual reads (representing a maximum of 0.009% of the full length sequence data) were discarded based upon the hypothesis that more abundant sequences are likely to be accurate biological sequences, whilst rare sequences are likely to contain sequencing errors or be due to PCR artefacts. Denoising reduced the total number of unique reads by an average of 32% (**Table 3**).

Clustering of denoised Ion Torrent reads at an identity of 97%, and therefore considering sequences within a 3% distance of each other to belong to a single OTU, generated between 16 and 28 OTUs for the 8 member mock population, and between 27 and 56 for the mock population known to contain 20 species depending on which genomic region was considered (**Table 4**). In all instances, the more variable ITS1 region [26] appeared to generate the most OTUs, and the least variable LSU region the fewest. Considering all samples, an average OTU to species ratio of 2.4±0.3 was observed. Further investigation revealed that the additional OTUs were the result of each single species being represented by more than one OTU, rather than additional “incorrect” species being identified. Each species was represented by a “major OTU” which accounted for approximately 99% of the reads assigned to that organism, and various significantly smaller OTUs which clustered separately due to the presence of insertion/deletion (INDEL) errors, often associated with homo-polymeric regions (please refer to File **S1** **and File S2**). In all cases, the major OTU showed the highest identity to the true biological sequence, with the additional smaller OTUs displaying base mis-matches due to INDELs. Such data are of great significance to metagenomic studies that commonly use OTU counts to infer species richness. Failure to carefully characterise the OTU to species ratio using mock communities (such as is presented herein) can therefore lead to vast under or over estimations of species diversity. Despite the publication of numerous NGS driven metagenomic studies (the vast majority concerning bacteria) [27], there is still a relative paucity of studies characterising such metrics in mock fungal communities.

**Table 4:** Characteristics of OTUs formed by the USEARCH algorithm at an identity of 0.97. Only OTUs with a minimum of 25 reads were retained.

Sample	8GM	8GM	8GM	20SM	20SM	20SM
**Region**	ITS1	ITS2	LSU	ITS1	ITS2	LSU
**OTU Minimum**	25	25	25	25	25	25
**OTUs**	28	24	16	56	44	27
**Perfect 0% Error**	10	7	8	33	16	14
**Good ≤2% Error**	13	8	3	19	21	5
**Noisy 3% Error**	3	6	1	2	3	5
**Contaminant**	0	0	3	2	3	1
**Other >3%**	2	3	1	0	1	2

In order to assess the accuracy of OTU formation, each OTU was compared to its closest true biological sequence using nucleotide blast (BLASTn), and mismatches between the two considered to be errors. OTUs were mapped to the NCBI database without restriction (i.e. this search was not limited to the fungi in the mock populations). OTUs were categorised as “Perfect” (0% errors), “Good” (≤2%), “Noisy” (3%), “Contaminant” (high identity to a true biological sequence not included within the mock population) or “Other” (≥3%). The vast majority of OTUs were categorised as “Perfect” or “Good” suggesting that highly accurate OTUs could be reconstructed from the denoised Ion Torrent sequencing data (**Table 4**).

**Figure 1:**
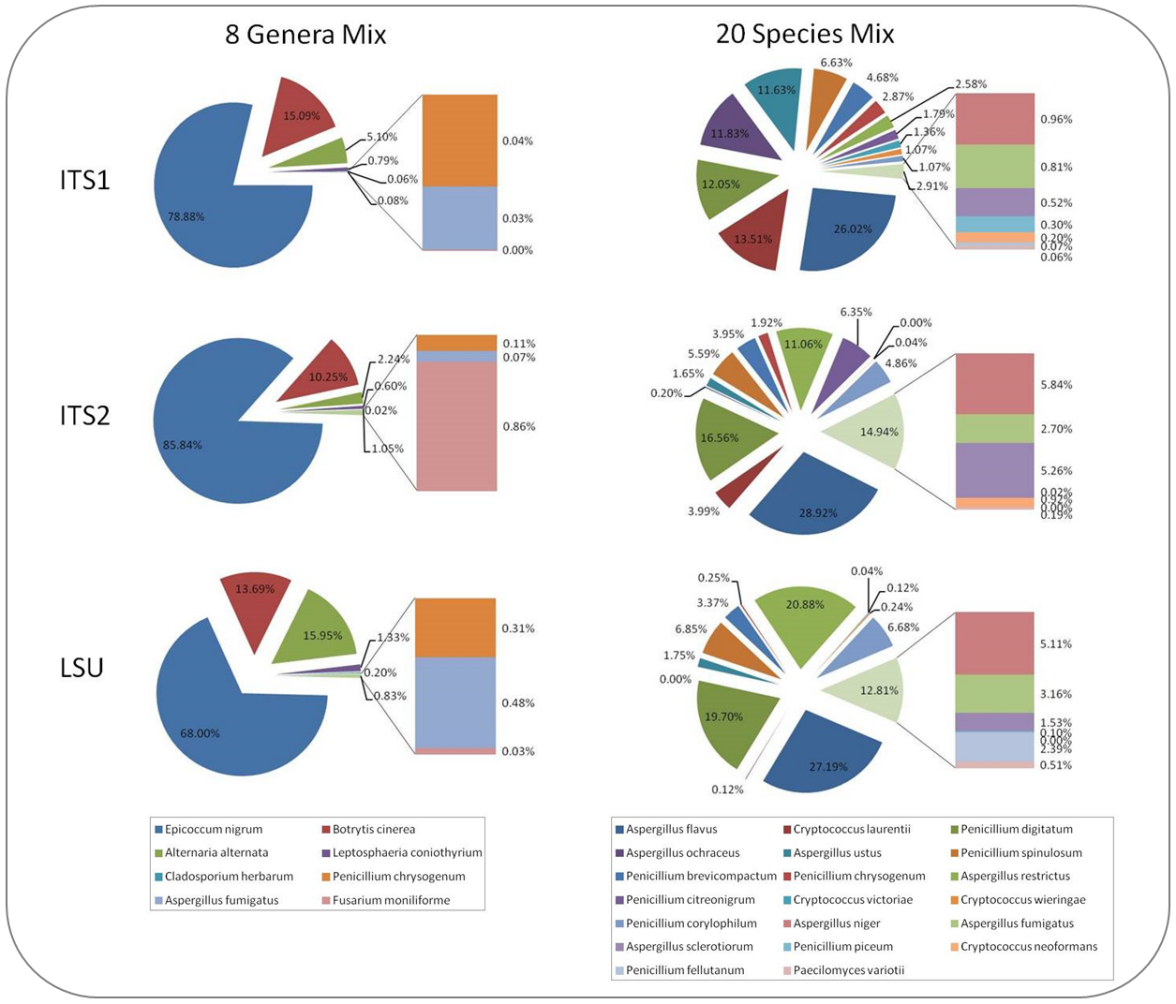
Read Distributions - the number of sequencing reads assigned to each species, expressed as a percentage of the total number of sequencing reads.

Finally, we determined whether the annotated OTUs (as above) accurately reflected the mock community members in the 8GM and 20SM samples based upon the three metagenomic regions considered. Each mock community member successfully represented by an OTU and the taxonomic classification afforded is recorded in (**Table 5**). In the vast majority of cases (23/28), an OTU representing each of the mock community members was present in the sequencing data from all three genomic regions. For 5 of the community members, representative OTUs were detected by 2 genomic regions (**Table 5**, designed “N”) but were absent from the sequencing data in the third. In the 8GM specimen, *Fusarium moniliforme* was not detected by ITS1, but was detected in both the ITS2 and LSU sequencing data. Subsequent sequence analysis revealed a mismatch between this organism and the forward ITS1 primer, preventing efficient amplification, and resulting in any sequencing data being lost at the adapter trimming stage (where a 100% primer match was required). In the 20SM specimen, *Cryptococcus victoriae* and *Penicillium fellutanum* were not detected by ITS2 whilst *Aspergillus ochraceus* and *Cryptococcus neoformans* were not detected by LSU. Amplification of the ITS2 region from *Cryptococcus victoriae* resulted in only partial reads of approximately 180 bases in length however no clear sequence composition issue was evident. Amplification of this region in *Penicillium fellutanum* also failed to generate full length reads however this was likely due to two homopolymeric regions consisting of 12 thymine residues and 7 cytosine residues respectively. Amplification products from the LSU region of *Aspergillus ochraceus* and *Cryptococcus neoformans* were also absent. Retrospective analysis of the raw sequencing data confirmed the absence of any amplification products from either species. It is known that *Cryptococcus neoformans* lacks one of the LSU priming sites preventing amplification. However, insufficient sequence information is available for *Aspergillus ochraceus* to determine whether this species also lacks one of the required priming sites. It is clear from these data that no one region was able to identify all 28 species that comprised the mock communities, further confirming our recommendation that a multi-region metagenomic approach should be taken where possible. An alternative strategy would be to utilise a single-marker, multi-primer approach, or even a multi-marker, multi-primer approach to increase the probability of detecting a given species. Following identification, the absolute number of reads mapping to each OTU (representing each community member) was determined and is shown in (**Figure 1**). Each species was included at an equal spore concentration within the two mock communities (**Table 3**) however the absolute number of reads mapping to each of the species varied by several orders of magnitude. For many species the proportion of reads attributed to that species within the mock communities was similar between the three regions, for example *Epicoccum nigrum* and *Aspergillus flavus* were the most abundant species in mock communities 8GM and 20SM respectively for all three regions, however for others abundance proportion varied more substantially (Figure 1).

**Table 5:** Taxonomic levels of discrimination afforded by each genomic region.

Sample (8GM)	8GM	8GM	8GM
Region	ITS1	ITS2	LSU
Detected/Expected	7/8	8/8	8/8
Alternaria alternata	G	O	G
Aspergillus fumigatus	S	S	S
Penicillium chrysogenum	G	G	G
Fusarium moniliforme	N	O	O
Leptosphaeria coniothyrium	S	S	O
Botrytis cinerea	G	G	G
Cladosporium herbarum	G	S	G
Epicoccum nigrum	S	O	O

Sample (20SM)	20SM	20SM	20SM
Region	ITS1	ITS2	LSU
Detected/Expected	20/20	18/20	18/20
Aspergillus flavus	Se	Se	Se
Aspergillus fumigatus	S	S	S
Aspergillus niger	Se	G	Se
Aspergillus ochraceus	Se	S	N
Aspergillus restrictus	S	S	Se
Aspergillus sclerotiorum	S	Se	Se
Aspergillus ustus	Se	Se	Se
Cryptococcus laurentii	S	S	S
Cryptococcus neoformans	S	S	N
Cryptococcus victoriae	G	N	S
Cryptococcus wieringae	S	S	S
Paecilomyces variotii	S	S	S
Penicillium brevicompactum	Se	Se	Se
Penicillium chrysogenum	G	G	G
Penicillium citreonigrum	Se	S	Se
Penicillium corylophilum	S	S	Se
Penicillium digitatum	S	S	S
Penicillium fellutanum	S	N	S
Penicillium piceum	S	S	S
Penicillium spinulosum	G	Se	Se

Species level - S, Section within genus level – Se, Genus level - G, Above Genus level - O, Not detected -N.

## Discussion

It is apparent from our data that proton release sequencing technology can deliver a qualitative assessment of the fungal members comprising a sample when carefully applied, and when paired with appropriate bioinformatic analysis. We used a multi-region approach to investigate any potential targeting bias which could arise when reliant on a single region. During the early years of barcoding the ITS region became the default marker for species level studies for most fungi; with the exception of the yeasts, where the LSU became the standard for identification [28], [29]. Given that yeasts and filamentous fungi are abundant in nearly all environments, including the human body, we chose to use the variable D1/D2 region of the LSU [16] and both ITS regions [15]. It is clear from our study that, whilst there were differences in read abundance proportion attributed to the different species between the three regions, the main concern was that no single region was able to identify all 28 species that comprised the mock communities; confirming our recommendation that a multi-region metagenomic approach should be taken where possible. Using a multi-region approach all 28 species could be identified, however, quantification is more difficult to achieve as indicated by the apparent discordance between spore number and read count.

Other studies have highlighted potential amplification biases dependent on the choice of ITS primers used, and an alternative to our multi-region approach is the multi-primer approach targeting different parts of the ITS region in parallel [29]. However, as the authors themselves point out, targeting the whole of ITS1 or ITS2 biases the results towards the Ascomycota; targeting only ITS1 may lead to a preferential amplification of ‘non-dikarya’ fungi, the dikarya being the subkingdom of fungi that includes Ascomycota and Basidiomycota which together represent about 80% of described fungal species. It is generally accepted that for the majority of the fungal Kingdom, ITS is the preferred barcode region, but that other supplementary regions may be needed [26]. LSU can often discriminate species independently, or more so combined with ITS; and for yeasts the D1/D2 region of LSU was adopted for characterising species before DNA barcoding became popular. Given that ITS sequences can be shared between different species such as *Cladosporium*, *Penicillium*, *Fusarium* and *Aspergillus*, four genera of both economical and clinical relevance, we opted to take a multi-region approach to include ITS1, ITS2 and the D1/D2 region of LSU. Data from other studies indicate that ITS and LSU perform very similarly as barcodes and that differences in these sequences correlate well with current species concepts [26]. Irrespective of approach, numerous primer combinations are reported within the literature [30], and it is important to determine the most optimal primer set or combination for each particular application.

This study used two mock fungal populations selected to address different questions. The 8GM reference mixture was used to reflect different genera that produce spores that are readily found in air samples [14]. It was utilised for this study as it reflects a diverse mixture of fungal genera that produce spores of different sizes and complexity, and simultaneously allow the direct comparison of the high throughput sequencing data generated in this study with data produced using the more traditional clone library preparation and Sanger sequencing approach. The 20SM reference mixture was chosen to comprise closely related species that produce single-celled spores of a more uniform size from clinically relevant fungal genera. The application of high throughput sequencing to assessing fungal diversity is of growing interest in studies of the human microbiome. ITS often has insufficient variation to unequivocally identify species in species-rich Ascomycete genera such as *Penicillium* [28]. 20SM includes several species of *Aspergillus*, *Penicillium* and *Cryptococcus* and one species of the genus *Paecilomyces*, a genus that is genetically close to both *Aspergillus* and *Penicillium*, and therefore better represents the types of species that might be found in a human microbiome study compared to 8GM. In addition, 20SM comprises species that produce single-celled spores of a fairly uniform size, unlike 8GM.

The fungal rDNA region, which is the molecular target of choice for fungal amplicon-based metagenomic studies given the existence of priming sites which are highly conserved across large groups of organisms, enables the amplification and detection of a wide range of taxa [6]. This fungal rDNA region is multicopy in nature, occurring numerous times throughout the fungal genome. Whilst multiple gene copies increase sensitivity by effectively amplifying the genomic signal from each fungal spore, it has been shown that copy number differs considerably between fungi, from tens to several hundred [31], [32] and will therefore impact upon the final number of sequencing reads attributed to each fungi. Attempts have been made to enumerate copy number in PCR studies by empirically determining the rDNA copy number relative to a single copy number gene [31], [32] and have confirmed that copy number does indeed vary by species. Dividing the number of sequencing reads by the copy number represents a plausible method of normalising next generation sequencing data. However, methods for the empirical calculation of rDNA copy number are only suitable for the detection and enumeration of a restricted set of fungi (where individual pure cultures are available) and are therefore presently unsuitable for the large scale metagenome studies arising as the result of next generation sequencing availability.

Differences in DNA extraction efficiency between the different fungal spore types, and the bias towards shorter amplicons inherent in PCR reactions, may also affect the number of sequencing reads attributed to each fungi [6]. Black and colleagues recently reported large variations in detectable target when PCR was conducted on DNA extracted from known quantities of spores [31] suggesting that DNA is not released from all spores with equal efficiency. Although differences in DNA extraction efficiency and PCR bias are likely to contribute less to differences in absolute read abundance than the effect of rDNA copy number variation, no comprehensive assessment in commonly encountered fungal spores has yet been conducted.

A further consideration is spore size and complexity, which is of particular relevance to the 8GM mock community formulated from fungi with a diverse range of spore sizes. The smaller sized, single celled spores from *Aspergillus fumigatus* and *Penicillium chrysogenum* were represented at much lower read numbers than the multicellular large spores from *Alternaria alternata*, *Leptosphaeria coniothyrium* and *Epicoccum nigrum* (Table 1 and Figure 1). The second mock population was, however, created solely from single celled spores of approximately 2 to 10 µm diameter, and the difference in read number from the least to most frequent was more than 300 fold for all three regions. The differences are unlikely to be due to any process inherent to the Ion Torrent library preparation and sequencing as an earlier study of the same 8GM mock community utilising traditional clone library preparation and Sanger sequencing found the same bias in terms of numbers of clones represented by each species (Pashley, Fairs et al., 2012; online supplement).

Next generation sequencing driven amplicon-based metagenomics have revolutionised the field of microbiology by providing a culture independent technique through which to identify and assess microbiological diversity. Furthermore, the availability of affordable “bench-top” sequencers has placed the ability to perform such studies in the hands of most laboratories. Here we have shown that data generated by proton release sequencing technology is suitable for use in amplicon driven metagenome studies. We have validated our approach using two mock fungal communities comprising 8 and 20 species respectively. Taking into account data generated from three individual genomic regions (ITS1 ITS2 LSU), we were able to construct OTUs representing each of the test species, and further, these OTUs were found to be highly accurate representations of true biological sequences. Finally, we considered potential barriers that require further investigation before this methodology can make the transition from qualitative (presence or absence) to quantitative (relative abundance of fungi within a complex population). Whilst these barriers affect all molecular applications targeting the rDNA region, including the traditional application of Sanger sequencing to PCR amplicons, the advent and availability of next generation sequencing technologies has refocused this issue.

## Supporting Information

File S1 **The number of OTUs corresponding to each species in the two controlled populations for ITS1, ITS2, and LSU.** Where more than one representative OTU was detected, the error type responsible for the additional OTUs is recorded in the remarks column: I – insertion, D – deletion, S – substitution, IH – insertion associated with homopolymer, DH, deletion associated with homopolymer.
(DOCX)

File S2 **Representative data demonstrating the presence of a “major OTU” for each species (OTU 1) accounting for the majority of reads, and various significantly smaller OTUs (OTUs 2–5 shown). Representative data is from the ITS1 region, 8GM.**
(DOCX)
