# Correction: Amplicon–Based Metagenomic Analysis of Mixed Fungal Samples Using Proton Release Amplicon Sequencing

**DOI:** 10.1371/journal.pone.0106021

**Published:** 2014-08-13

**Authors:** The PLOS ONE Staff

The second and third authors, Catherine H. Pashley and Timothy W. Gant, should be noted as contributing equally to this work.

Additionally, Catherine H. Pashley should not be noted as a corresponding author. Daniel P Tonge is the sole corresponding author for this article.

## References

[1] Tonge DP, Pashley CH, Gant TW (2014) Amplicon –Based Metagenomic Analysis of Mixed Fungal Samples Using Proton Release Amplicon Sequencing. PLoS ONE 9(4): e93849 doi:10.1371/journal.pone.0093849 24728005 10.1371/journal.pone.0093849 PMC3984086

