# Supplementary material for: Amplicon –Based Metagenomic Analysis of Mixed Fungal Samples Using Proton Release Amplicon Sequencing
Source: PLoS One. 2014 Apr 11;9(4):e93849. doi: 10.1371/journal.pone.0093849 (PMC3984086; doi:10.1371/journal.pone.0093849)
Supplement: File S1 — The number of OTUs corresponding to each species in the two controlled populations for ITS1, ITS2, and LSU. Where more than one representative OTU was detected, the error type responsible for the additional OTUs is recorded in the remarks column: I – insertion, D – deletion, S – substitution, IH – insertion associated with homopolymer, DH, deletion associated with homopolymer. (DOCX) [file pone.0093849.s001.docx]

Supplementary Data


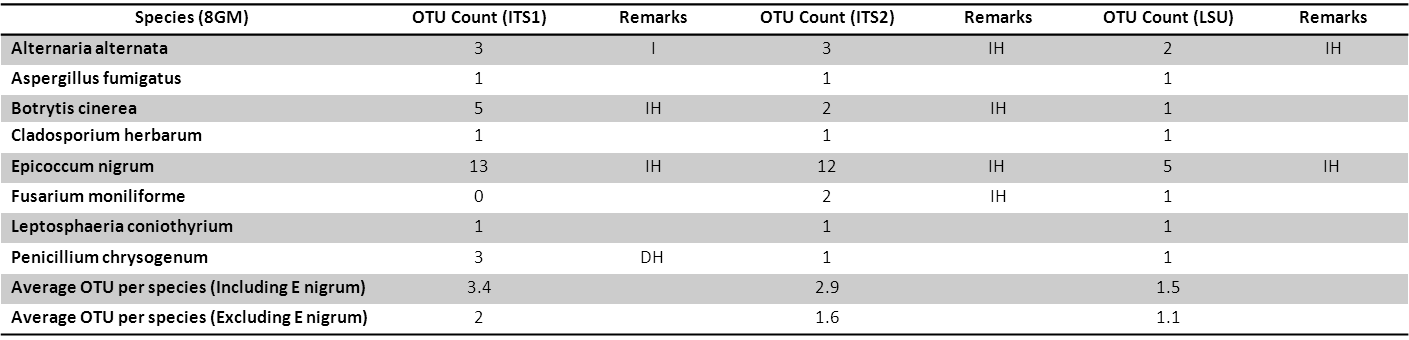


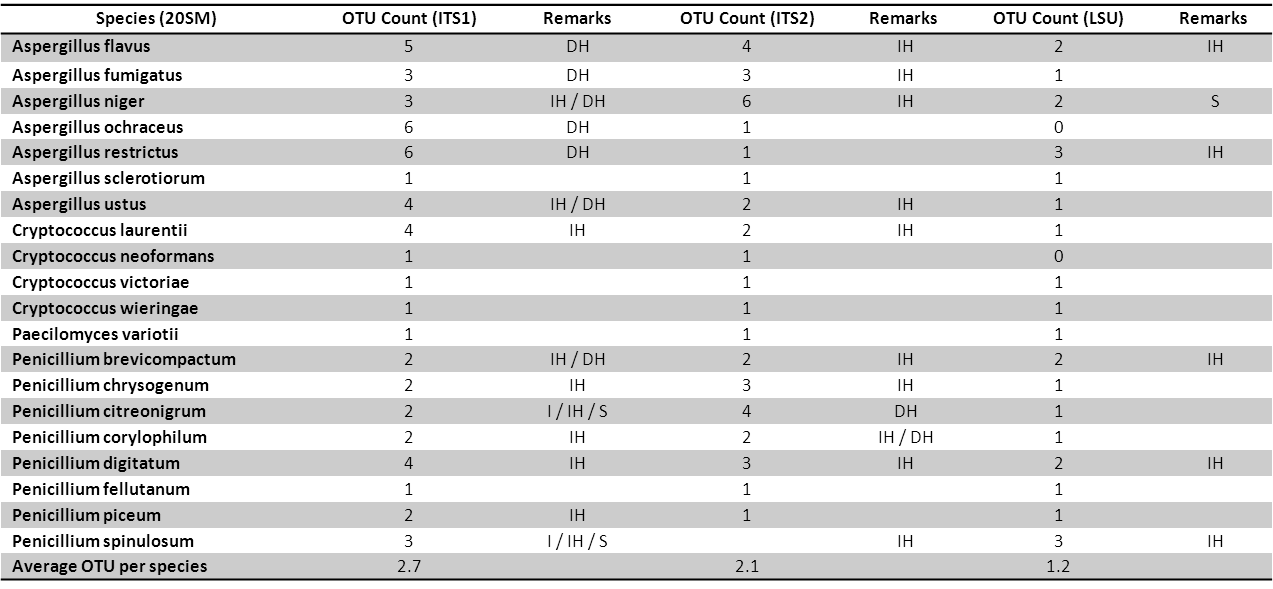


**S1** – The number of OTUs corresponding to each species in the two controlled populations for ITS1, ITS2, and LSU. Where more than one representative OTU was detected, the error type responsible for the additional OTUs is recorded in the remarks column: I – insertion, D – deletion, S – substitution, IH – insertion associated with homopolymer, DH, deletion associated with homopolymer.
