# Supplementary material for: Amplicon –Based Metagenomic Analysis of Mixed Fungal Samples Using Proton Release Amplicon Sequencing
Source: PLoS One. 2014 Apr 11;9(4):e93849. doi: 10.1371/journal.pone.0093849 (PMC3984086; doi:10.1371/journal.pone.0093849)
Supplement: File S2 — Representative data demonstrating the presence of a “major OTU” for each species (OTU 1) accounting for the majority of reads, and various significantly smaller OTUs (OTUs 2–5 shown). Representative data is from the ITS1 region, 8GM. (DOCX) [file pone.0093849.s002.docx]

**S2** – Representative data demonstrating the presence of a “major OTU" for each species (OTU 1) accounting for the majority of reads, and various significantly smaller OTUs (OTUs 2 – 5 shown). Representative data is from the ITS1 region, 8GM.
